# Preferred and actual retirement age of oral and maxillofacial surgeons aged 55 and older in the Netherlands: a longitudinal study from 2003 to 2016

**DOI:** 10.1186/s12960-018-0288-6

**Published:** 2018-05-30

**Authors:** Joost C. L. den Boer, Steven A. Zijderveld, Josef J. M. Bruers

**Affiliations:** 1 Department of Research, Royal Dutch Dental Association (KNMT), Utrecht, the Netherlands; 2 Department of Oral and Maxillofacial Surgery, St Antonius Hospital, Nieuwegein, the Netherlands; 3 Department of Oral and Maxillofacial Surgery, St Antonius Hospital, Utrecht, the Netherlands; 4 Academic Centre for Dentistry Amsterdam (ACTA), University of Amsterdam and Vrije Universiteit, Department of Social Dentistry and Behavioural Sciences, Amsterdam, the Netherlands

**Keywords:** Workforce planning, Retirement planning, Preferred retirement age, Actual retirement age, Oral and maxillofacial surgeons

## Abstract

**Background:**

In workforce planning for oral and maxillofacial surgeons in the Netherlands, it is important to plan timely, as these dental specialists are required to earn both medical and dental degrees. An important factor to take into account in workforce planning is the outflow of the profession through retirement. In the workforce planning in the Netherlands, it was assumed that retirement plans are a predictor for the actual moment of retirement. The purpose of this study was to investigate this assumption.

**Methods:**

A standardised survey to investigate the work activity and retirement plans of oral and maxillofacial surgeons was conducted seven times between 2003 and 2016. With some minor variations, in every edition, all oral and maxillofacial surgeons aged 55 years and older who did not indicate to be retired in an earlier edition were invited to participate. The data of all seven editions was analysed to investigate what factors influence the actual retirement age. For the analyses of the data, ANOVA and linear regression were employed.

**Results:**

The response rate was at least 80% in all editions. For all editions combined, 185 surgeons were invited one or more times, of whom 170 responded at least once. Between 2003 and 2016, the mean preferred retirement age increased from 63.7 to 66.7. Two thirds of the respondents who participated in more than one edition had revised their preferred retirement age upwards. Regarding the difference between preferred and actual retirement age, 45% of the oral and maxillofacial surgeons retired at a higher age than originally preferred and another 14% was still working at the age the originally preferred to retire. Linear regression shows that preferred retirement age is associated with sex and the number of working hours and that actual retirement age is associated with preferred retirement age, earlier preference to decrease working hours and working in non-academic hospitals.

**Conclusion:**

Altogether, it seems that in this group the preferred retirement age has some predictive value, but the oral and maxillofacial surgeons tend to retire at a higher age than they originally preferred to.

## Background

Between 2000 and 2016, the mean retirement age of employees in the Netherlands increased from 61.0 to 64.4 years [1]. This increase applied to employees in all distinct sectors and industries. For health care professionals, the mean retirement age rose from 60.4 years in 2006 to 63.9 years in 2016. Likewise, the mean retirement age of physician specialists, of whom 61% are self-employed and thus not employees, has increased from 61.3 years in 2000 to 63.2 years in 2013 [2]. The largest increase occurred between 2006 and 2011 [3]. Although the increase of the mean retirement age seems to have slowed in recent years, it is not inconceivable that the retirement age will rise again. In 2013, the Dutch government implemented the ‘general old age pension and pension target age act’ (*wet verhoging AOW- en pensioenrichtleeftijd*). Under this law, the legal retirement age will gradually increase from 65 to 67 years in 2021 and possibly further onwards. The legal retirement age, or the age an individual is eligible for a state pension, will be matched with life expectancy as of 2023 [4]. Life expectancy is expected to increase in the coming years [5].

Deciding on retirement is a complex process in which many factors are involved [6]. The identified factors are financial situation, health, presumed health, working conditions, attitude towards work and retirement and the work situation of one’s spouse or partner [7–18]. Feldman and Beehr [19] distinguish three phases in the retirement decision-making process, which are not completely separate and can take place simultaneously. In the first phase—imagining the future—the possibilities of retirement are considered. In the second phase—assessing the past—an individual thinks about when it is time to leave his or her job. In the third phase—transitioning into retirement in the present—the considerations of the first two phases are converted into concrete plans, and action is taken. Solem et al. [20] also distinguish three levels of retirement planning that differ in firmness: considerations, preferences and decisions. They find significant correlations between retirement preferences and actual retirement age as well as between retirement decisions and actual retirement age. They note that this correlation is particularly true for preferences and decisions to retire at a normative age. Furunes et al. [21] conclude, based on a longitudinal, qualitative panel study, employees do not change their retirement plans completely in the last phases of their career, but they do adjust their plans slightly.

Starting from a sufficiently large workforce, an increase in the mean retirement age of an occupational group can have consequences for the succeeding generation, as it can create a surplus of professionals if inflow does not proportionally decrease. On the other hand, if a too high estimation of the retirement age is taken into account in workforce planning, a shortage can emerge. Both a surplus and shortage of doctors can cause unwanted effects. A surplus can lead to inefficiency, overtreatment, unemployment and outmigration. A shortage can result in waitlists and quality loss because the workload is too high and the available time is insufficient to deliver the best quality care [22, 23]. Therefore, an inaccurate estimation of the outflow of professionals can lead to unwanted fluctuations in the number of professionals available for practice [24–26].

A main objective of workforce planning is to prevent unwanted fluctuations [27]. This is especially important in health care, because the education of health care professionals is expensive and takes a long time [28]. In the Netherlands, education costs even more for oral and maxillofacial surgeons (OMFS), as candidate OMFS are required to earn both medical and dental degrees [29]. Both of these educations have 6-year curricula. It is possible to get exemptions from courses, but it is still necessary to plan ahead for the duration of one’s specialisation, which is 4 years. Therefore, in workforce planning for OMFS, it is important to adequately consider the expected developments in the demand for and supply of specialists [30]. Therefore, it is necessary to understand when the incumbent professional group will retire. In many countries, workforce planners face difficulties in formulating expectations regarding the retirement of doctors [28]. Additionally, social, cultural, epidemiological and demographic developments should be taken into account [31].

Workforce planning for medical and dental professionals in the Netherlands is exercised by the Capacity Body (CO), which was established in 1999. The main task of the CO is to assess estimates of the future capacity of health care professionals. The CO collects empirical data on the supply of health professionals (e.g. the number of professionals, length of working week and task distribution). Moreover, the CO collects data on the demand for care, including epidemiological information, number of patients and changes in patients’ expectations and behaviour. The CO has different chambers and working groups for different areas of health care, including one for dental specialists, OMFS and orthodontists. Educators and health insurance companies also participate in this working group. Members of the Dutch Society of Oral and Maxillofacial Surgery (NVMKA), the Dutch scientific association for OMFS, and the Royal Dutch Dental Association (KNMT), the main professional association of dentists, OMFS and orthodontists in the Netherlands, represent OMFS. The NVMKA and the KNMT share a common interest and have made contributions to this working group for several decades. In 1994, a committee was established to facilitate this. This committee experienced a lack of adequate information on the work activity and intended retirement plans of OMFS. These plans were seen as an indicator for the actual retirement age, as OMFS are self-employed and therefore not bound to the legal retirement age. Therefore, in 2003, a standardised survey was established to investigate the work activity and retirement plans of older OMFS. This survey was repeated several times between 2003 and 2016.

In this manuscript, data from all seven editions of the survey are analysed to answer the following questions. How have the preferred and actual retirement ages of older OMFS developed between 2003 and 2016? To what extent do older OMFS adjust their retirement plans in the last years of their professional careers? What characteristics affect the desired and actual retirement age of older OMFS?

## Methods

In 1996 and 2002, the KNMT conducted two surveys on retirement and retirement plans of OMFS. Both surveys were conducted in different populations of OMFS, and each used a different questionnaire. In 2003, a new questionnaire was developed, which was used in all subsequent surveys. In this questionnaire, OMFS retirement is defined as stopping patient treatment. This is a change of career or employment later in life, which is one of the eight definitions of retirement Denton and Spencer distinguish [32]. The other seven definitions are non-participation in the labour force, reduction in hours worked and/or earnings, hours worked or earnings below a minimum, receipt of retirement income, leaving one’s main employer, self-assessed retirement and any combination of the former.

Originally, the survey was to be conducted every other year. However, the sixth edition was postponed by several governmental measures [33]. These measures became effective in January 2015 and obliged hospitals, physician specialists and OMFS to develop a new organisational structure [34]. In 2013, OMFS were not expected to oversee the consequences of these measures on their work and retirement situations. As one of the objectives of the survey was to investigate retirement plans, it was considered inefficient to carry out the survey in a time of such uncertainty. Therefore, the sixth edition of the survey was postponed to 2014 in the hope that OMFS would be able to better assess the consequences of the changes.

In contrast to the questionnaire, the research population changed to some extent between editions. In 2003, the survey started with OMFS aged 55 years and older, and in the two subsequent editions, OMFS 50 and older were included. After 2007, the population was once again restricted to those 55 years and older. Until 2016, there was no maximum age. In the last edition, the maximum was set at 79 years. In previous editions, all OMFS aged 80 years and older were retired. Therefore, it was considered undesirable to bother these OMFS with a questionnaire that most likely did not apply to them. In all the editions, the population was invited to participate with the exception of OMFS who indicated being retired in previous editions. For this purpose, the details of all OMFS within the selected age group were retrieved from the dentist database of the KNMT. In this continuously updated database, all dentists and dental specialists are registered, including non-members and retired professionals. The retirees were excluded based on their own indication in an earlier edition of the survey. In all editions, written questionnaires were sent to the OMFS by mail and at least two reminders were sent. However, in 2014, OMFS were given the opportunity to fill in a web-based questionnaire. In this survey, OMFS were asked about their retirement plans.

In all editions of the survey, the response rate was 80% or greater (Table 1). For all editions combined, 185 OMFS were invited to participate in the survey at least once, of whom 170 (92%) responded one or more times. More specifically, 37 OMFS participated once (of whom 22 were eligible for participation for the first time in the last edition and 6 indicated to be retired in the first edition), 40 OMFS participated twice, 29 OMFS three times, 24 OMFS four times, 21 OMFS five times, 15 OMFS six times and four OMFS seven times. Almost all respondents (98%) were male; 2% were female. Furthermore, 26% were born in 1944 or earlier, 22% between 1945 and 1949, 25% between 1950 and 1954 and 27% in 1955 or later. The majority of participants were based in the western Netherlands, 17% in the south, 17% in the east and 12% in the north. Overall, 15 OMFS (8%) did not participate in any edition of the survey. With regard to the individual characteristics mentioned above, the group of non-respondents showed no statistical significant differences (*p* < 0.05) from the 170 OMFS who participated in one or more editions.

**Table 1:** Research population and response rates of all editions of the retirement survey among Dutch OMFS aged 55 and older

Edition	Year	Research population^a^	Number of respondents	Response percentage	Number of respondents still working
1	2003	61	54	89	48
2	2005	64	55	86	50
3	2007	65	52	80	52
4	2009	76	76	100	68
5	2011	89	78	88	76
6	2014	123	106	86	78
7	2016	123	102	83	79
	Total^b^	185	170	92	

^a^All OMFS aged 55 and older were invited to participate in the survey, with the exception of OMFS who indicated being retired in a previous edition. In 2016, OMFS aged 80 years and older were not invited

^b^All OMFS were invited to participate in the survey at least once. OMFS who responded to at least one edition of the survey are considered respondents

The data from the different editions of the survey were merged according to corresponding respondent numbers and were analysed using IBM SPSS Statistics version 24. For the bivariate analyses, ANOVA was used, and for multivariate analyses, stepwise linear regression (minimum F to enter is .050 and minimum F to remove is .100) was employed. In the description of the results, a significance level of 0.05 was used.

## Results

**Fig. 1:**
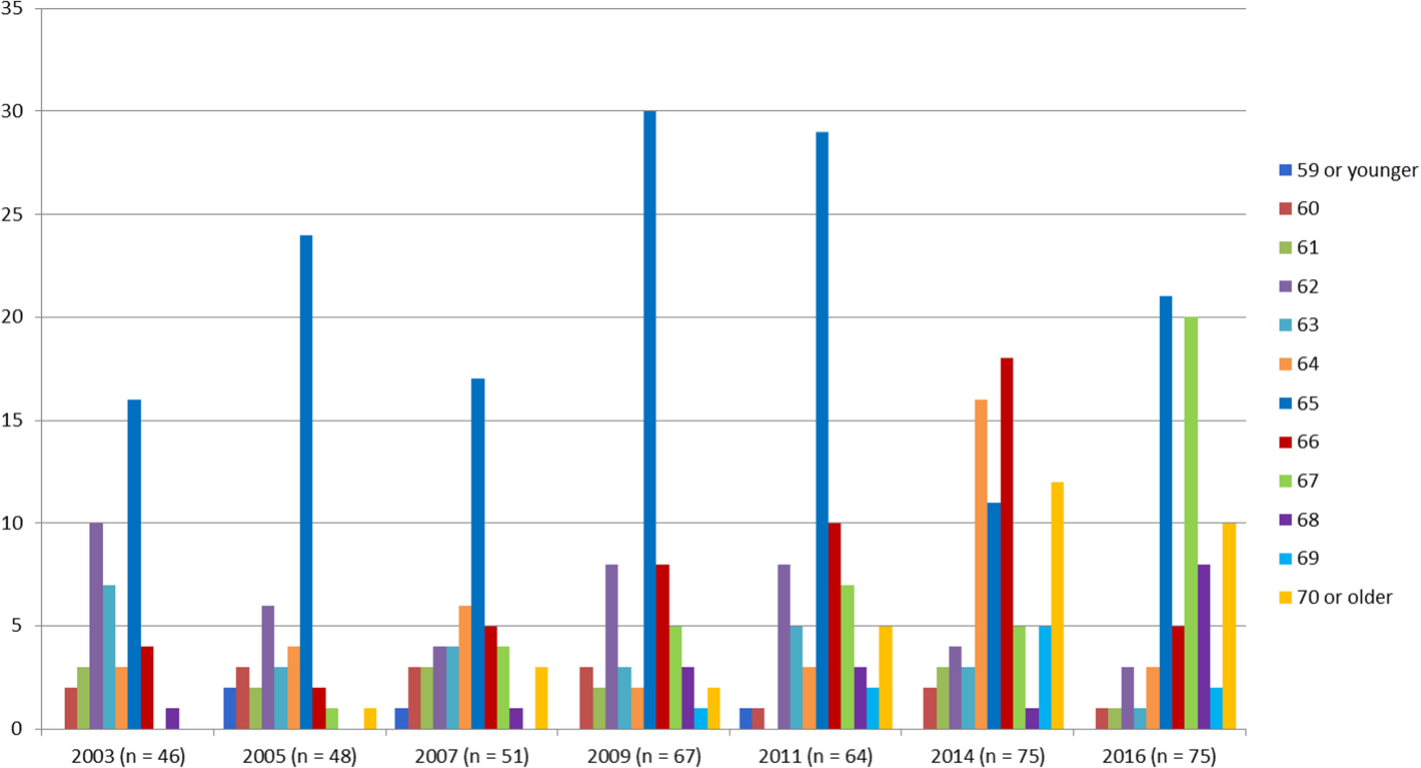
Counts of the preferred retirement age of Dutch OMFS 55 years and older between 2003 and 2016

**Fig. 2:**
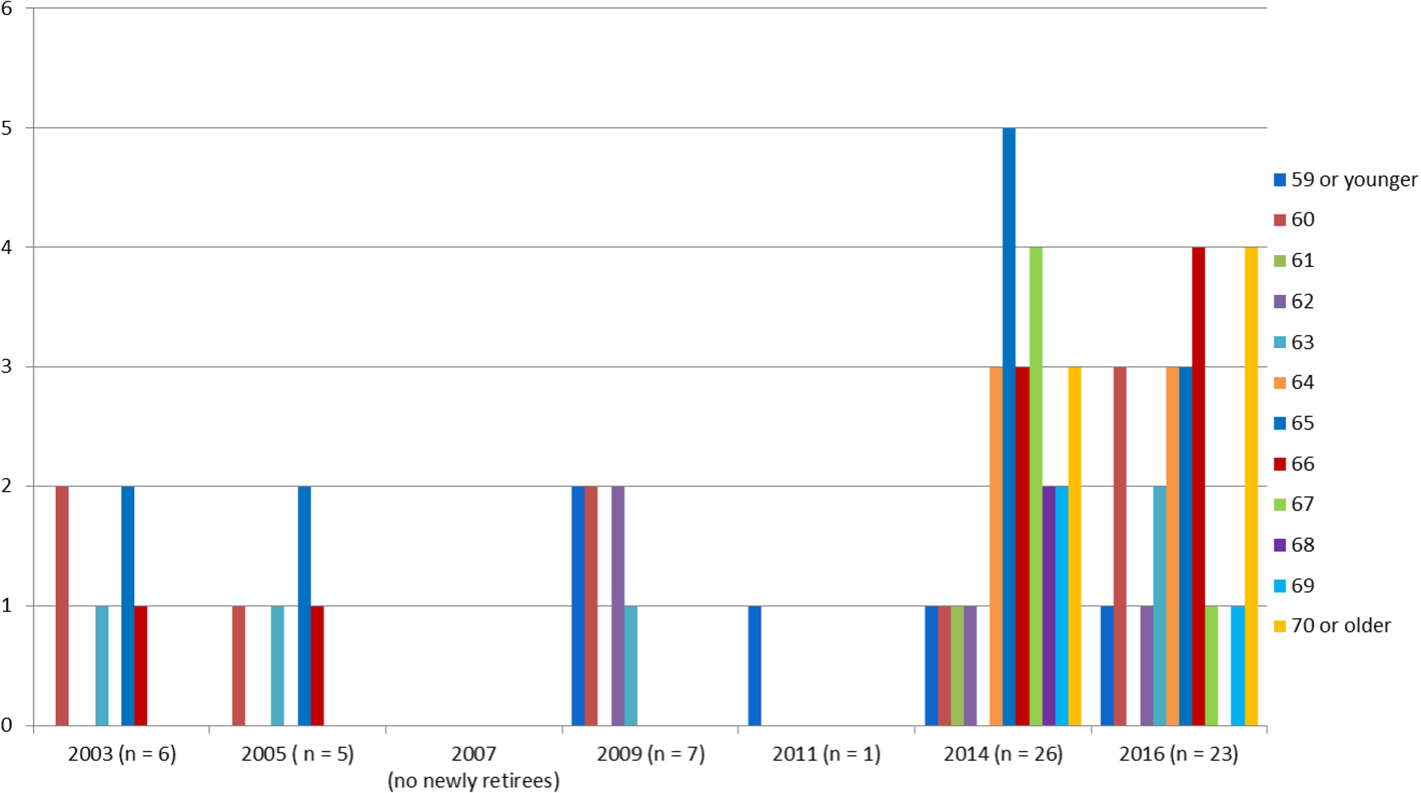
Counts of the actual retirement age of newly retired Dutch OMFS 55 years and older between 2003 and 2016

In Fig. 1, the counts of the preferred retirement age of the OMFS are shown. The mean preferred retirement age increased from 63.7 in 2003 to 66.7. Figure 2 shows the counts of the actual retirement age of the participants who retired. As OMFS who have retired before are excluded from the research population, these are all newly retirees.

Table 2 shows that 78 (67%) of the 116 OMFS who reported a preferred retirement age in more than one edition had revised their preferred retirement age upwards, 24 (21%) did not change their preference and 14 (12%) adjusted their preferred retirement age downwards. The mean adjustment was + 2.1 years.

**Table 2:** Difference between preferred retirement age of Dutch OMFS of 55 years and older who participated in two or more editions of the retirement survey

	Number	Percentage	Difference (years)
Adjusted preferred retirement age downwards	14	12	− 2.0
Did not adjust preferred retirement age	24	21	0.0
Adjusted preferred retirement age upwards	78	67	3.5
Total	116	100	2.1

If an OMFS indicated a preferred retirement age in more than two editions, the difference between them was calculated. The difference between preferred retirement ages in different editions is expressed in years. A positive score indicates the OMFS adjusted the preferred retirement age upwards, and a negative score indicates the OMFS adjusted the preferred retirement age downwards

In Table 3, the differences between preferred and actual retirement age are shown. If an OMFS revised his or her preferred retirement age, the earliest answer is used. The majority of the 51 pensioned OMFS retired later than originally intended, 11 retired at the age they initially preferred and 11 retired at a younger age. The mean difference was + 1.4 years. Furthermore, 14 OMFS were still active after the age they originally preferred to retire.

**Table 3:** Difference between preferred and actual retirement age of Dutch OMFS of 55 years and older who retired between participation two editions of the survey

	Number	Percentage	Difference (years)^a^
Retired younger than first preferred retirement age	11	17	− 3.4
Retired at first preferred retirement age	11	17	0.0
Retired older than first preferred retirement age	29	45	3.7
Total	51	100	1.4

^a^The difference between preferred and actual retirement age is expressed in years. A positive score indicates the OMFS retired older than first preferred, and a negative score indicates the OMFS retired younger than first preferred

Table 4 shows the results of the bivariate analyses, in which individual characteristics are related to preferred and actual retirement age. The table indicates that OMFS aged 55 to 59 and OMFS aged 60 to 64 prefer to retire at a younger age than both OMFS aged 65 and older. Moreover, OMFS who work in academic hospitals prefer to retire at an older age than OMFS who work in other settings, and OMFS who work seven dayparts (morning, afternoon or evening) a week or less prefer to retire at younger age than OMFS who work eight or more dayparts per week. Further, OMFS who prefer to retire at age 59 or younger actually retired at younger age than OMFS who prefer to retire at an older age. OMFS who prefer to decrease their amount of working hours retired at a younger age than OMFS who did not, and OMFS who worked in non-academic hospitals retired at an older age than their colleagues who did not.

**Table 4:** Mean preferred and actual retirement age of Dutch OMFS aged 55 years and older, in relation to individual characteristics

	Preferred retirement age (*N*)^a^		Actual retirement age (*N*)	
Sex				
Male	64.0 (143)		64.4 (67)	
Female	66.0 (4)			
Age^a^		*		
59 years or younger	64.0 (126)		Not applicable	
60–64 years	64.4 (20)		Not applicable	
65 years or older	70.0 (1)		Not applicable	
Region located^a^				
North	64.5 (20)		64.3 (7)	
East	64.4 (25)		64.8 (8)	
South	63.5 (25)		64.3 (12)	
West	64.1 (73)		64.6 (38)	
Working in an academic hospital^a,b^		*		
Yes	64.7 (41)		64.0 (15)	
No	63.8 (105)		65.1 (39)	
Working in a non-academic hospital^a,b^				*
Yes	64.0 (119)		65.4 (43)	
No	64.5 (27)		62.5 (11)	
Working in another setting^a,b^				
Yes	64.2 (42)		64.1 (18)	
No	64.0 (104)		65.2 (38)	
Dayparts per week working^a^		***		
7 or less	62.9 (34)		64.1 (20)	
8–9	64.0 (43)		65.0 (10)	
10 or more	64.7 (69)		64.8 (21)	
Prefer to decrease working hours^a^				*
Yes	63.8 (40)		62.4 (14)	
No	64.2 (106)		65.7 (40)	
Decreased working hours^c^				
Yes	Not applicable		65.0 (22)	
No	Not applicable		64.7 (32)	
Preferred retirement age^a^				*
59 years or younger	Not applicable		58.0 (2)	
60–64 years	Not applicable		64.5 (31)	
65 years or older	Not applicable		65.3 (8)	
Total	64.1 (147)		64.4 (67)	

*p < 0.05; **p < 0.01; ***p < 0.001

^a^If an OMFS revised his or her preference or if the actual situation changed in between editions, the first answer is used. For age and location, the situation at the first participation is used

^b^It is possible that an OMFS works in different hospitals and/or settings

^c^Between participations in different editions of the OMFS retirement survey. An OMFS who indicated to work 10 dayparts per week in the first edition of the survey and eight dayparts per week in the second edition, decreased working hours by two dayparts

The results of the multivariate analyses are included in Tables 5 and 6. Table 5 shows that female OMFS prefer to retire when they are almost 5 months older (0.39 years) than their male colleagues do. Furthermore, the more hours per week OMFS work, the older they prefer to retire.

**Table 5:** Association between preferred retirement age and individual characteristics of Dutch OMFS aged 55 years and older (*n* = 146)

	*B*	Standard error	*β*	Tolerance	VIF	*p*
Constant	60.87	0.73				
Female	0.39	0.08	0.16	1.00	1.00	0.04*
Dayparts per week working	2.17	1.06	0.36	1.00	1.00	0.00***
*R*^2^ = 0.15						
Durbin-Watson = 1.63						

The following individual characteristics were considered in the analyses: sex, age at first participation, region located, work setting, dayparts per week working and preference to decrease working hours. Only the statistically significant associations are displayed. If an OMFS revised his or her preference or if the actual situation changed in between editions, the first answer is used

*p < 0.05; **p < 0.01; ***p < 0.001

**Table 6:** Association between actual retirement age and individual characteristics of Dutch OMFS aged 55 years and older who retired between editions of the retirement survey (*n* = 51)

	*B*	Standard error	*β*	Tolerance	VIF	*p*
Constant	24.33	13.39				
Working in a non-academic hospital^a^	3.61	1.17	0.37	0.95	1.05	0.00***
Prefer to decrease working hours^b^	− 3.41	1.08	− 0.38	0.95	1.05	0.00***
Preferred retirement age^c^	0.61	0.21	0.34	0.99	1.01	0.01**
*R*^2^ = 0.34						
Durbin-Watson = 1.79						

The following individual characteristics were considered in the analyses: sex, region located, work setting, dayparts per week working, decreased working hours and preferred retirement age. Only the statistically significant associations are displayed

*p < 0.05; **p < 0.01; ***p < 0.001

^a^Dummy variable. It is possible that an OMFS works in different hospitals and/or settings

^b^This indicates that an OMFS suggested in an earlier edition of the survey to prefer to decrease working hours

^c^If an OMFS revised his or her preference or if the actual situation changed in between editions, the first answer is used

With regard to the actual age OMFS retired, Table 6 indicates that the higher the preferred retirement age, the higher the actual retirement age. For every increase of the preferred retirement age by 1 year, the actual retirement age, which basically is higher, increases more than 7 months (0.61 years). Furthermore, OMFS who indicated preferring to decrease their working hours in an earlier edition retired at a younger age than OMFS who did not prefer this. In addition, OMFS who used to work in non-academic hospitals retired at an older age than OMFS who only worked in academic hospitals and/or other kinds of practices. The explanatory power of the first model is rather low (*R*^2^ = 0.15). In the model for actual retirement age, the explanatory power is somewhat higher (*R*^2^ = 0.33).

## Discussion

Between 2003 and 2016, the preferred retirement age of ‘older’ OMFS gradually increased. On the other hand, the increase in actual retirement age is less clear. This presumably has to do with the small size of the research population. In spite of the fact that in all editions the entire population of older OMFS was solicited for the survey and that the response rates were high, the actual number of participants is still rather low. In total, 170 OMFS participated in one or more surveys, 51 of whom retired at some point. When the total number of respondents in a category is this low, the influence of outlier values can be significant. These outliers can be explained by several factors, such as data recording or entry errors, sampling errors, environmental conditions and motivated misreporting [35]. Due to the small sample size and the limited number of variables, it is possible to check all data for entry errors and make corrections. Sampling errors cannot be the cause of deviant values in this survey, as the entire population was solicited. Non-response bias can be a determinant of errors [36]. However, the available individual background characteristics do not show differences between the 170 respondents and the 15 non-respondents. The possibility that environmental conditions influenced the results was recognised. After all, one edition of the survey was postponed due the effects of governmental measures regarding the work situations of OMFS. Obviously, it is possible that deviant values are caused by intended or unintended misreporting or by the fact that there are OMFS that have considerably different retirement plans than their colleagues. Altogether, we tentatively conclude that the preferred and actual retirement age have increased since 2003.

Between 2003 and 2016, the research population increased from 61 to 123, due to the age composition of the population of OMFS in the Netherlands. Data from the KNMT dentist database suggest that the population of OMFS 55 and older will increase further until at least 2020. The increase of the population can partly explain the increase in number of OMFS who indicated to be retired in the last two editions of the survey. Other factors of possible influence are the fact that the 2014 editions followed an edition in which a rather small number OMFS indicated to be retired; the interval between the fifth and sixth edition was a year longer than usual and an increase of retirement age. The latter can be the result of the increase of the legal retirement age, although OMFS are self-employed and therefore are not bound to the legal retirement age.

The results of the seven editions of the survey indicate that OMFS adjust their retirement plans in the last years of their professional careers. Most OMFS who did so adjusted their preferred retirement age upwards. Furthermore, OMFS who have retired generally stopped treating patients at an older age than they originally indicated as their preferred retirement age. This should be taken into account when using the preferred retirement age for capacity planning to avoid high costs for training a surplus of OMFS. In the survey, unretirement was not taken into account, as retired OMFS were excluded for the following editions. Although unretirement is not uncommon, economic factors play an important role in the decision to unretire [37–39]. These factors are not applicable in the Netherlands [40]. Furthermore, the post-retirement jobs differ from the pre-retirement jobs and appear to be less demanding [38]. Therefore, it was concluded that unretirement as an OMFS is rare in the Netherlands.

The rationale for the survey was to gain insight into the outflow of OMFS. Solem et al. [20] conclude that the preferred retirement age is a particularly powerful predictor of actual retirement age when it corresponds with a normative retirement age. For many years, in the Dutch pension system, the normative retirement age was 65 years. However, the normative retirement age will increase to 67 years in 2021 and will probably increase more thereafter. From 2003 to 2011, the mode of preferred retirement age of older OMFS was 65; in 2014, it was 67. In 2016, the mode was 65 again, but there were two peaks in preferred retirement age: 21 OMFS planned to stop at 65, 20 planned to stop at 67 and all other ages were mentioned by eight OMFS or fewer. It is expected that the mean pension age of OMFS will increase in the coming years.

Due to the rationale of the survey, the questionnaire was short. As a result, the available data did not give overwhelming insights into the characteristics and factors that affect preferred and actual retirement ages. It seems, however, that the preferred retirement age is a valid and reliable predictive factor for actual retirement age.

Even in this small, homogenous group of highly educated health care professionals, it seems that more factors than the ones included in the survey affect retirement age. From the literature, it is evident that financial situation, health, presumed health, working conditions, attitude towards work and retirement and the work situation of one’s spouse or partner are important factors [7–18]. An extensive questionnaire covering all these factors did not fit within the scope of the survey, which was designed to give a quick view of retirement plans without further investigation into the context. To accomplish this, a high response rate was desired. Jepson et al. [41] demonstrate that questionnaire length affected the response rate for a mailed survey of generalist physicians; shorter questionnaires got higher response rates. This short questionnaire indeed accomplished high response rates of 80% or greater.

Another factor that was not investigated is the reason for the adjustment of retirement age. Adjustments can be voluntary after reconsideration or involuntary, for example, when poor health prevents an OMFS from continuing work [42]. As some conditions can appear abrupt, this can immediately influence the predictive power of the preferred retirement age for that individual. Therefore, the actual retirement age can only be forecasted by preferences to some extent.

Despite these observations, the survey data helped KNMT and NVMKA in developing a point of view on future training places. As said, OMFS are a very specific group of highly educated professionals. Therefore, the results cannot be generalised for all occupational groups. On the other hand, all physicians are highly educated professionals and in the Netherlands many medical specialists work in settings comparable to the working settings of OMFS.

## Conclusions

Altogether, it seems that for older OMFS the preferred retirement age has predictive value for outflow from the profession in the coming years. It should be noted that OMFS tend to retire at a somewhat higher age than they originally prefer to.

## Abbreviations

ACTA: Academic Centre for Dentistry Amsterdam
CO: Capacity Body
KNMT: Royal Dutch Dental Association
NVMKA: Dutch Society of Oral and Maxillofacial Surgery
OMFS: Oral and maxillofacial surgeons
